# Somatosensory cortex participates in the consolidation of human motor memory

**DOI:** 10.1371/journal.pbio.3000469

**Published:** 2019-10-15

**Authors:** Neeraj Kumar, Timothy F. Manning, David J. Ostry

**Affiliations:** 1 McGill University, Montreal, Canada; 2 Indian Institute of Technology Gandhinagar, Gandhinagar, India; 3 Haskins Laboratories, New Haven, Connecticut, United States of America; UCSF, UNITED STATES

## Abstract

Newly learned motor skills are initially labile and then consolidated to permit retention. The circuits that enable the consolidation of motor memories remain uncertain. Most work to date has focused on primary motor cortex, and although there is ample evidence of learning-related plasticity in motor cortex, direct evidence for its involvement in memory consolidation is limited. Learning-related plasticity is also observed in somatosensory cortex, and accordingly, it may also be involved in memory consolidation. Here, by using transcranial magnetic stimulation (TMS) to block consolidation, we report the first direct evidence that plasticity in somatosensory cortex participates in the consolidation of motor memory. Participants made movements to targets while a robot applied forces to the hand to alter somatosensory feedback. Immediately following adaptation, continuous theta-burst transcranial magnetic stimulation (cTBS) was delivered to block retention; then, following a 24-hour delay, which would normally permit consolidation, we assessed whether there was an impairment. It was found that when mechanical loads were introduced gradually to engage implicit learning processes, suppression of somatosensory cortex following training almost entirely eliminated retention. In contrast, cTBS to motor cortex following learning had little effect on retention at all; retention following cTBS to motor cortex was not different than following sham TMS stimulation. We confirmed that cTBS to somatosensory cortex interfered with normal sensory function and that it blocked motor memory consolidation and not the ability to retrieve a consolidated motor memory. In conclusion, the findings are consistent with the hypothesis that in adaptation learning, somatosensory cortex rather than motor cortex is involved in the consolidation of motor memory.

## Abstract 2

This study provides direct evidence that learning-related changes to somatosensory cortex are involved in the consolidation of human motor memory. Specifically, transcranial magnetic stimulation applied to somatosensory cortex following motor learning blocks motor memory consolidation, whereas sham stimulation and suppression of activity in primary motor cortex have no adverse effect on retention.

## Introduction

One of the most striking abilities of the famous patient HM is that he was able to learn and retain novel motor skills even though he lost all capacity to form other long-term memories following bilateral medial temporal lobe resection [1–3]. The neuroanatomical basis of this spared ability remains uncertain. Although there is extensive evidence of motor cortex involvement in learning [4–12], direct evidence of its involvement in motor memory consolidation is weak [13–16] (see below), transient [17–19], or absent altogether [20]. An alternative possibility, which we pursue here, is that somatosensory cortex participates in memory consolidation. It is known that somatic plasticity occurs in conjunction with newly learned movements [21]. This takes the form of changes to both sensed limb position [22, 23] and somatic acuity [24, 25]. Somatosensory cortex has also been shown to be involved in error processing directly related to learning [26]. Plasticity in somatosensory cortex may reflect the acquisition and storage of newly learned sensory states (new somatic targets) that guide subsequent movements. If this is the case, then the suppression or disruption of somatosensory cortex activity following learning should block memory consolidation and adversely affect the retention of motor memory.

Targeted brain stimulation can be used to suppress individual cortical regions in order to assess their involvement in memory consolidation. A number of studies that have focused on motor memory consolidation have used transcranial magnetic stimulation (TMS), which was delivered to motor cortex at the end of each trial during adaptation learning [13–15]. Adverse effects of TMS on retention have been identified, but in each case, they have been limited in magnitude. In other work, TMS was applied to motor cortex immediately prior to learning [16]. When tested 24 hours later, these participants showed less retention than controls, but there was still substantial retention overall. However, stimulation during or before learning complicates the identification of brain areas involved in retention, since it may also affect the learning process itself in ways that are not easily measurable and might impede consolidation as a consequence. An alternative approach is to disrupt candidate structures following the completion of training (to block consolidation) and then, following a delay that would normally permit consolidation, assess whether there is an impairment in retention. A small number of studies have taken this approach. It has been reported that repetitive transcranial magnetic stimulation (rTMS) to primary motor cortex following motor learning substantially disrupted retention of a simple ballistic movement task [19], but it did not alter retention of a more complex motor task involving altered dynamics [20]. However, the disruption observed with simple movements may be due to testing for retention immediately following TMS. When additional time was added following TMS to permit overnight consolidation of learning, there was little evidence of interference with retention [17, 18], suggesting that TMS had effects on motor cortex that were transient but did not prevent the eventual consolidation of learning.

In the present study, continuous theta-burst transcranial magnetic stimulation (cTBS) [27, 28] was applied either to motor cortex or to somatosensory cortex immediately following force-field adaptation with the goal of blocking motor memory consolidation. It has been shown in other work that cTBS suppresses the excitability of cortex in both areas of the brain [27–30]. In both somatosensory and motor cortex, we tested for the consolidation of learning using an experimental manipulation in which the force field was introduced gradually to minimize conscious awareness of the perturbation and associated explicit learning strategies [15, 31, 32]. We also tested for consolidation when the load was introduced abruptly with the aim of engaging both explicit strategies and implicit processes in the subsequent adaptation [32–34]. After adaptation and then cTBS, there was a 24-hour delay followed by tests of motor memory retention. It was found that following adaptation to a gradually introduced perturbation, cTBS to somatosensory cortex almost entirely eliminated retention, which indicates that somatosensory cortex participates in the consolidation of motor memory. A control study, in which cTBS was applied to somatosensory cortex following memory consolidation, showed no effects on retention and confirmed that cTBS following learning blocked memory consolidation rather than retrieval. In contrast, the disruption of retention following the abrupt introduction of load was only partial, which suggests that areas of the brain other than somatosensory cortex are involved in the consolidation of more explicit components of motor memory. Primary motor cortex did not appear to be involved in the consolidation of either implicit or explicit motor learning, as indicated by the finding that cTBS to primary motor cortex following adaptation to either gradual or abruptly introduced loads had effects on retention that were no different than sham TMS. Thus, in force-field adaptation, a circuit involving somatosensory but not motor cortex is involved in the initial consolidation of motor memory. The retention of explicit strategies in motor learning appears to be dependent on neither somatosensory nor motor cortex.

## Results

**Fig 1:**
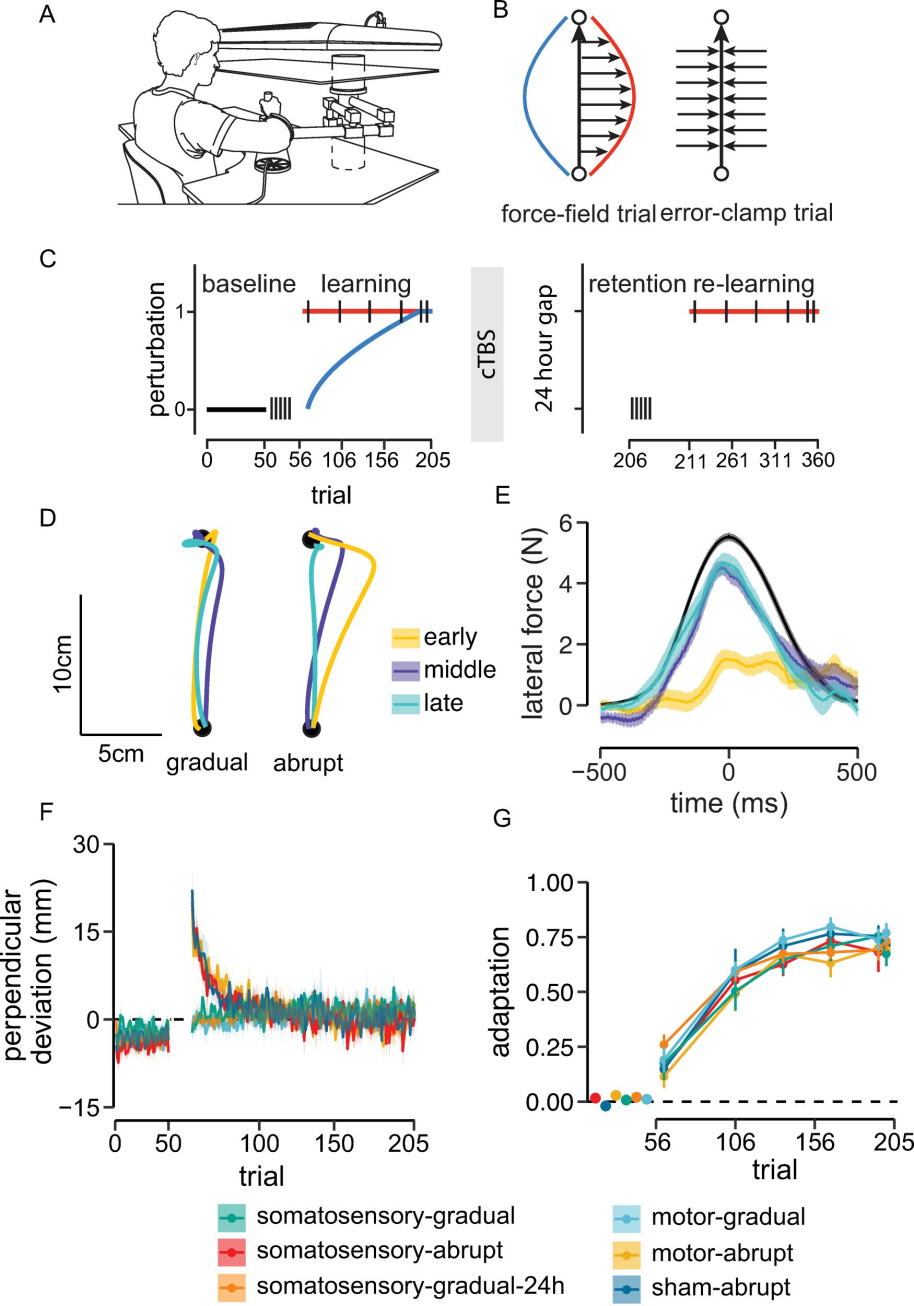
Experimental procedure and learning performance during day 1 training. (A) Sketch of the setup used to display the start and target positions, and a participant holding the robotic manipulandum. Sketch is adapted from van Vugt and colleagues [36]. (B) Schematic of force-field and error-clamp trials. The red curve exemplifies the force applied by the robot arm, and the blue curve illustrates the ideal compensatory force profile. In error-clamp trials, the robot constrained the hand movement to a straight line to the target. Forces applied to the channel walls serve a measure of learning and retention. (C) Schematic of various phases of the experimental procedure. Participants in somatosensory-gradual, motor-gradual, and somatosensory-gradual-24h groups trained with a gradually introduced force field on day 1 (blue). Participants in the somatosensory-abrupt, motor-gradual, and sham-abrupt groups trained with an abruptly introduced force field on day 1 (red). Relearning on day 2 involved an abruptly introduced perturbation for all the groups. Black vertical bars represent the position of error-clamp trials. (D) Representative hand paths in early (yellow), middle (violet), and late (magenta) phases of adaptation to a gradual and abrupt perturbation. (E) Lateral force profiles produced by participants in different phases (early, middle, late) of the initial training session. The force profile in black represents the averaged ideal force profile needed to completely compensate for the applied perturbation. (F) Learning curves showing mean PD across trials for each experimental condition. All the groups reached similar performance levels by the end of the initial training session, and PDs were close to zero. (G) Change in the adaptation coefficient during learning (linear regression of the ideal force onto the actual force during error-clamp trials). Each group reached similar adaptation levels by the end of training. Shaded regions and error bars represent ±SEM across participants. Data used to generate the figures in (E), (F), and (G) can be found in S1 Data. cTBS, continuous theta-burst transcranial magnetic stimulation; PD, perpendicular deviation.

Participants in these studies held the handle of a robotic manipulandum (Fig 1A) and made reaching movements in a velocity-dependent force field [35] (Fig 1B). This was followed immediately by cTBS stimulation to either motor or somatosensory cortex to block motor memory consolidation. Participants returned 24 hours later to assess retention of learning. In the initial training session, the force field was introduced either gradually over trials or all at once at the beginning of training. The progressive increase in force-field strength in the gradual condition was designed to minimize participants’ awareness of its presence and engage implicit learning processes. In contrast, the abruptly introduced force field served to engage both explicit strategies and implicit processes in adaptation. Tests of retention after 24 hours involved error-clamp trials in which movement was confined to a simulated mechanical channel (Fig 1B) followed by a retraining session with abrupt introduction of load. Fig 1C provides a summary of the different phases of the experiment and the conditions. In the results that follow, the different conditions—for example, somatosensory-gradual or motor-abrupt—are named with the convention that the first term describes the area of stimulation and the second term refers to the motor learning paradigm.

Movement trajectories in the gradual condition were close to straight throughout learning (Fig 1D), and the perpendicular deviation (PD) from a straight line was close to zero (Fig 1E). In contrast, in the abrupt condition, movement trajectories were curved upon initial exposure to the force-field perturbation (Fig 1D) and gradually became straighter, as reflected in a reduction in PD with practice (Fig 1F). In the initial training phase, prior to cTBS, the initial PD, averaged over the first 10 trials, differed among experimental conditions (F_[5,54]_ = 30.19, *p* < 0.001, ω_p_^2^ = 0.71). There was no significant difference in initial performance for individuals that trained with abruptly introduced loads (somatosensory-abrupt, motor-abrupt, and sham-abrupt, *p* > 0.9), nor did the rate of learning differ across the groups that learned with an abrupt perturbation (F_[2,27]_ = 0.47, *p* = 0.62, ω_p_^2^ = −0.04). Similarly, there was no difference initially for individuals that trained with gradual loads (somatosensory-gradual and somatosensory-gradual-24h, *p* > 0.9). Initial PDs for gradual groups were near to zero and were significantly different from those for individuals in abrupt conditions (*p* < 0.001).

Initial learning, prior to cTBS, was accompanied by a progressive increase in force in error-clamp trials (Fig 1E). As learning progressed, the force applied to the channel walls approached the force needed to fully compensate for the load applied by the robot (Fig 1G). In statistical tests, by the end of the first day of training, participants showed significant force compensation on channel trials in comparison to baseline forces measured before the learning session (F_[1,54]_ = 1,442.72, *p* < 0.001, η_p_^2^ = 0.96) and reached approximately 70% of the full force compensation (Fig 1G). There was no significant difference in adaptation coefficients for the different experimental conditions at the end of training (F_[5,54]_ = 0.26, *p* = 0.93, ω_p_^2^ = −0.06). By the end of the initial session, all the participants reached the same level of applied force regardless of whether the perturbation was introduced gradually or abruptly (*p* > 0.95).

**Fig 2:**
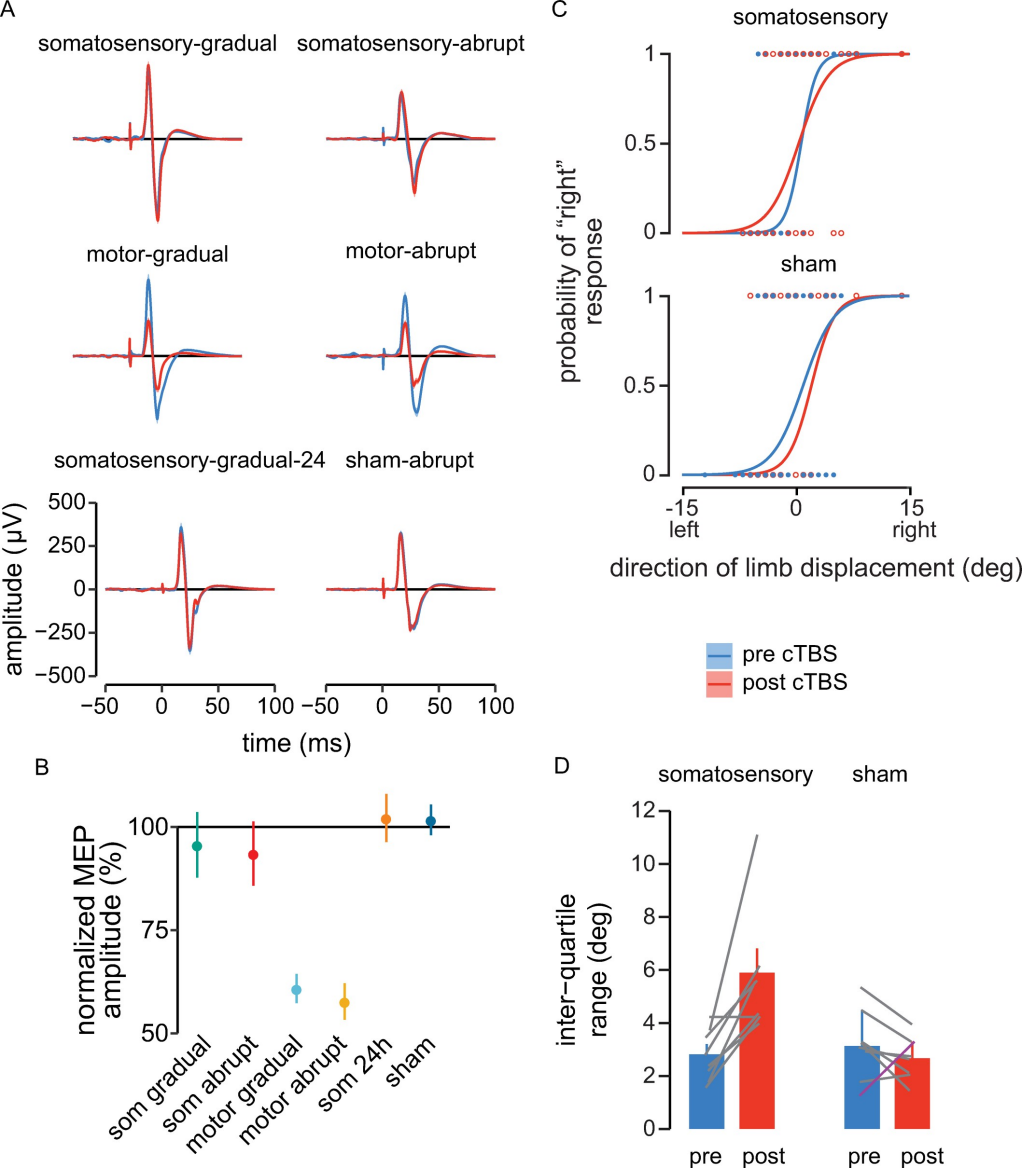
Excitability of motor cortex is not affected by cTBS over somatosensory (“som”) cortex, whereas cTBS over motor cortex suppresses its excitability. cTBS to somatosensory cortex decreases perceptual acuity. (A) Mean time series of MEPs recorded from biceps brachii pre- (blue) and post-cTBS (red) from a representative participant. The TMS pulse occurs at t = 0 ms. The shaded regions are ±SEM across 20 MEPs. (B) Mean change in amplitude of MEPs recorded post-cTBS (expressed as a percentage of pre-cTBS MEPs). Error bars give standard error across participants. (C) Psychometric fit to perceived limb position, showing performance of a representative participant before and after cTBS and sham stimulation. (D) Interquartile range pre- and post stimulation. Participants showed a decrease in perceptual acuity after suppression of somatosensory cortex. Error bars show standard error across participants. Data used to generate the figures in (B) and (D) can be found in S2 Data. cTBS, continuous theta-burst transcranial magnetic stimulation; MEP, motor-evoked potential; TMS, transcranial magnetic stimulation.

cTBS was applied following the initial learning session to block the consolidation of motor memory. Motor-evoked potentials (MEPs) measured before and after cTBS are shown in Fig 2A for a representative participants. Participants showed significant changes in MEPs after cTBS (F_[5,54]_ = 12.32, *p* < 0.001, ω_p_^2^ = 0.48). Pairwise comparisons showed that when cTBS was applied over somatosensory cortex, there was no effect on the excitability of motor cortex (somatosensory-gradual, somatosensory-gradual-24h, and somatosensory-abrupt groups, *p* > 0.9, Fig 2A and 2B), whereas a decrease in MEP magnitude was observed when stimulation was applied directly over motor cortex (motor-abrupt, motor-gradual, *p* < 0.002 Fig 2A and 2B). These results argue against the possibility of indirect inhibitory effects on motor cortex due to somatosensory cortex stimulation.

**Fig 3:**
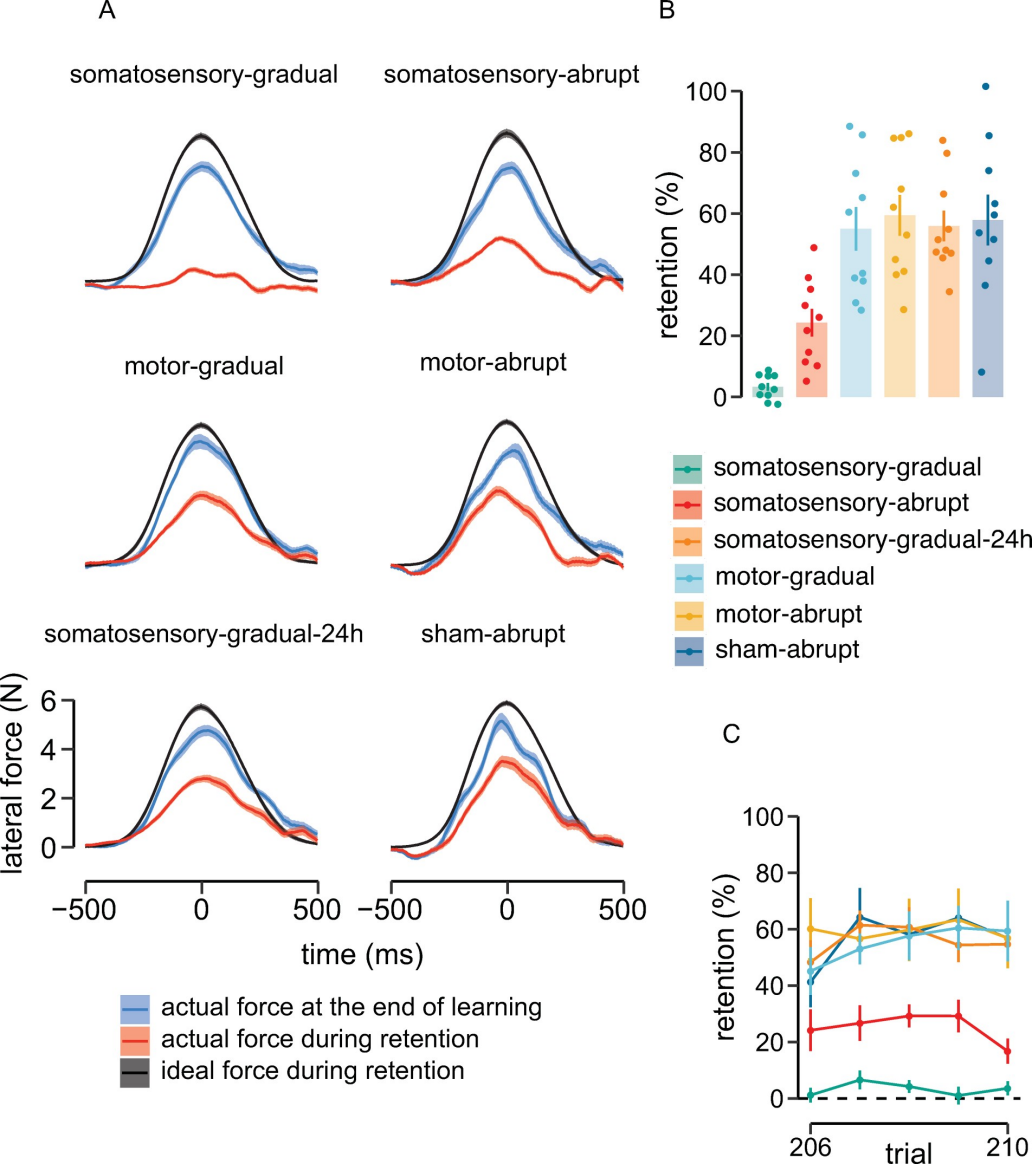
Suppression of somatosensory cortex using cTBS disrupts the consolidation of motor memory. (A) Mean force profiles (in red) produced by participants in each group during error-clamp trials in a retention session 24 hours after initial training. The force profile in black represents the expected force profile if participants showed full retention of initial learning. The force profile in blue is the actual force produced at the end of initial learning. When loads were introduced gradually (to engage implicit learning mechanisms), suppression of somatosensory cortex (somatosensory-gradual) completely eliminated retention as indicated by near-zero force levels in the retention test. When loads were introduced abruptly (somatosensory-abrupt), in order to engage both explicit and implicit mechanisms, cTBS to somatosensory cortex led to partial retention. cTBS did not interfere with consolidation when applied to motor cortex after learning either with a gradual (motor-gradual) or abrupt (motor-abrupt) onset of perturbation. Participants in these conditions produced forces comparable to those in the sham condition. The suppression of somatosensory cortex 24 hours after consolidation of initial learning (somatosensory-gradual-24h) had no effect on retention (forces were comparable to those in the sham condition), which indicates that cTBS to somatosensory cortex does not block the retrieval of previously consolidated memories. (B) Percent retention (amount of retention after 24 hours divided by the amount of adaptation at the end of initial training) assessed through error-clamp trials. Dots represent the mean retention (5 trials) for each participant, and bars indicate the average across participants for each group. (C) Percent retention across trials for each experimental condition. There is no change in retention across the five error-clamp trials. Shaded regions and error bars show standard errors across participants. Data used to generate the figures in (A), (B), and (C) can be found in S3 Data. cTBS, continuous theta-burst transcranial magnetic stimulation.

The role of motor and somatosensory cortex in motor memory consolidation was assessed by using error-clamp trials 24 hours after the initial learning session. Fig 3A shows participants’ actual force at the end of learning (blue), the ideal force that would be observed in the case of full retention (black), and the actually observed force on the channel walls during the retention test (red). It can be seen that in comparison to individuals that received sham stimulation and showed partial retention 24 hours later, individuals in the somatosensory-gradual group showed greatly impaired retention, as reflected by the near-zero forces in error-clamp trials (Fig 3A). In contrast, individuals in the somatosensory-abrupt group showed partial retention (Fig 3A). Individuals in the motor-abrupt and motor-gradual groups, in which cTBS was applied to primary motor cortex following learning, produced forces that were similar to those of sham-stimulation individuals, indicating that the suppression of motor cortex following learning does not block motor memory consolidation.

To test for the possibility that poor retention in the somatosensory-gradual condition was due to a memory retrieval failure rather than an effect of cTBS on memory consolidation, a further test was run in which a 24-hour delay was introduced following motor learning (gradual load) to permit consolidation (somatosensory-gradual-24h). Following this delay, cTBS was applied to somatosensory cortex. Retention and relearning tests were conducted approximately 4 hours later to determine whether cTBS to somatosensory cortex interferes with the retrieval of previously consolidated motor memories. As seen in Fig 3, these participants showed normal retention in channel trials (similar to motor cortex and sham) and normal patterns of relearning, which rules out the possibility that cTBS to somatosensory cortex results in a memory retrieval failure.

In statistical tests, there were reliable differences between experimental conditions in the percentage of initial learning that was retained at retest (amount of retention on day 2, as assessed using error-clamp trials relative to the amount of adaptation at the end of learning on day 1) (F_[5,54]_ = 15.54, *p* < 0.001, ω_p_^2^ = 0.54, Fig 3B). Pairwise comparisons showed that retention for the somatosensory-gradual group was significantly less than that observed in each of the other groups (*p* < 0.005). Retention for the two motor cortex stimulation conditions (abrupt and gradual) did not differ (*p* > 0.9), nor did they differ from the sham-stimulation group (*p* > 0.9). However, each of these conditions showed significantly greater retention than participants that received somatosensory stimulation after abrupt learning (*p* < 0.002). In turn, retention for participants in somatosensory-abrupt condition was significantly greater than that in the somatosensory-gradual condition (*p* = 0.015). No changes in performance were observed over the set of five error-clamp trials (F_[5,50]_ = 1.06, *p* = 0.39, η_p_^2^ = 0.1), indicating there was neither forgetting nor relearning during the retention test (Fig 3C).

**Fig 4:**
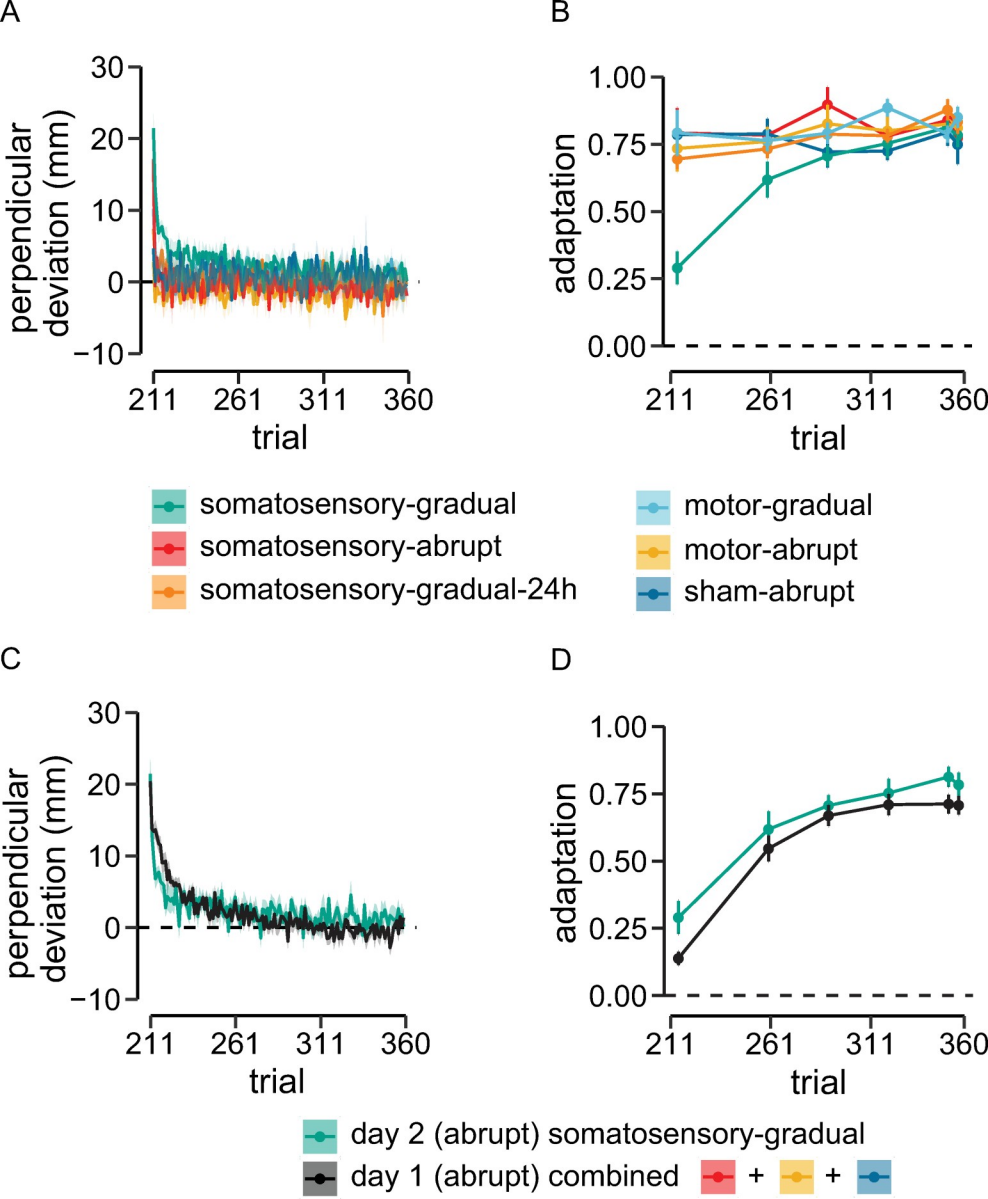
Suppression of somatosensory cortex causes participants to behave like naïve learners when reexposed to the perturbation. (A) There is rapid relearning in groups that had shown retention in the preceding error-clamp trials (motor-gradual, motor-abrupt, somatosensory-abrupt, somatosensory-gradual-24, sham-abrupt). Participants in somatosensory-gradual group showed slower relearning. (B) For participants shown in (A), from the beginning of the relearning session, adaptation coefficients are similar to those at the end of initial learning on day 1. (C) Relearning following cTBS for somatosensory-gradual group overlaid on averaged initial learning performance of naïve participants learning an abruptly introduced load. Note that all conditions shown here involved learning an abruptly introduced perturbation. (D) Overlay of adaptation coefficients during the relearning session for the somatosensory-gradual group with those of naïve participants. Performance of somatosensory-gradual group in the relearning session was similar to naïve learners, indicating the participation of somatosensory cortex in motor memory consolidation. Shaded regions and error bars give standard errors across participants. Data used to generate the figures in (A), (B), (C), and (D) can be found in S4 Data. cTBS, continuous theta-burst transcranial magnetic stimulation.

To further assess the contribution of motor and somatosensory cortex to retention of learning, retention tests using channel trials were followed by a relearning session in which all participants were exposed to an abrupt force-field perturbation. It can be seen that movement error in the relearning session, as assessed using PD, decreased relatively rapidly for somatosensory-abrupt participants (Fig 4A, red line) and more slowly for the somatosensory-gradual participants (cyan). Individuals in all other groups (motor-abrupt, motor-gradual, somatosensory-gradual-24h, and sham-abrupt) had PDs that were close to zero throughout relearning trials (Fig 4A, all other colors).

A similar pattern was obtained in the error-clamp trials that were interspersed in the relearning session. The adaptation coefficients for the first error-clamp trial differed among experimental conditions (F_[5,54]_ = 7.79, *p* < 0.001, ω_p_^2^ = 0.36, Fig 4B) and were significantly less for the somatosensory-gradual group (cyan) than for each of the other groups (*p* < 0.002), indicating a lack of retention. By the end of relearning session, all groups reached same level of adaptation (F_[5,54]_ = 0.52, *p* = 0.76, ω_p_^2^ = −0.04).

Fig 4C shows a comparison of kinematic error during relearning trials for individuals in the somatosensory-gradual condition (cyan) along with averaged kinematic data for participants that experienced an abrupt force field on day 1 (black). Participants in the somatosensory-gradual condition, when faced with an abruptly introduced load in the relearning session on day 2, showed an initial PD (F_[3,36]_ = 1.69, *p* = 0.18, ω_p_^2^ = 0.05) and rate of relearning that were not different from that of participants that trained with abrupt perturbations on day 1 (F_[3,36]_ = 1.21, *p* = 0.32, ω_p_^2^ = 0.02; Fig 4C). Further, as was observed in the relearning kinematic data, the pattern of error-clamp trials during relearning for participants in the somatosensory-gradual condition (cyan) was not significantly different from that of naïve participants learning the abrupt force field for the first time on day 1 (F_[15,180]_ = 0.39, *p* > 0.9, η_p_^2^ = 0.03; Fig 4D).

In a control study, to verify that cTBS blocked memory consolidation through an effect on the somatosensory system, we tested for changes to measures of sensed limb position that resulted from cTBS stimulation. Fig 2C shows a psychometric fit to a representative participant’s responses to the displacement of the limb to the left or right of the body midline. Consistent with the idea that the present cTBS protocol disrupts normal perceptual processing in somatosensory cortex, the difference between pre- and poststimulation measures of sensitivity is different for participants in the somatosensory and sham-stimulation groups (F_[1,14]_ = 14.91, *p* = 0.0017, ω_p_^2^ = 0.47). It is seen that in comparison to measures taken before cTBS (red), there is a reduction following stimulation in perceptual acuity of limb position (blue). This is reflected in an increase in the interquartile range of the psychometric function (*p* = 0.001, Fig 2D). Individuals that receive sham stimulation show no change (*p* = 0.861, Fig 2D). This test was repeated after removing the outlier participant in the somatosensory group of Fig 2D. The increase in interquartile range remained significantly higher with this individual removed (*p* = 0.002).

In a final test, we assessed the relationship between initial learning and retention. We computed a correlation between end-of-learning performance as measured on day 1 (last two error-clamp trials) and retention on day 2 (average performance over all five error-clamp trials) relative to each group’s average retention. By expressing retention scores relative to the mean retention in each condition, we were able to examine the relationship between learning and retention, separate from cTBS effects. The analysis revealed a positive correlation (R = 0.42, *p* < 0.001), indicating that participants that learned more initially showed better retention.

## Discussion

The consolidation of motor memory makes possible the retention of newly learned movements. The possible involvement of motor and somatosensory cortex in consolidation was assessed by applying cTBS following learning, with the goal of blocking consolidation and negatively affecting retention. Primary motor cortex was targeted based on the idea that newly learned movements require updated feedforward motor commands that may be encoded in frontal motor areas. Somatosensory cortex was tested on the assumption that motor memory entails the stabilization of learning-related somatic plasticity, that is, of updated sensory states that form the targets (or expected outcomes) of learned movements. The motor learning task involved force-field adaptation, in which the load was applied either gradually, with the goal of only engaging implicit learning processes, or abruptly to enable both implicit processes and explicit strategies. It was found that cTBS to somatosensory cortex impaired somatic perception. When applied following force-field learning with gradual loads, cTBS to somatosensory cortex greatly reduced retention, both as measured in force-channel trials and in measures of relearning. It was confirmed that cTBS blocked memory consolidation rather than retrieval. When a delay was added following learning to permit consolidation, cTBS to somatosensory cortex had little effect on retention. These results are consistent with the participation of somatosensory cortex in a circuit involved in the consolidation of motor learning. Partial retention was observed if the stimulation to somatosensory cortex was applied after training with an abrupt load onset, which suggests that areas other than somatosensory cortex encode explicit strategies involved in motor learning. Motor cortex participants trained with gradually or abruptly introduced loads to test for its possible role in the consolidation of either implicit or explicit learning. However, cTBS to motor cortex had little effect on retention. Measures of retention and relearning were no different than those obtained following sham TMS. The absence of an effect on consolidation for motor cortex participants also shows that the effects of cTBS are specific; namely, they suppress retention only when delivered to somatosensory cortex.

Although there is no evidence to date suggesting the involvement of somatosensory cortex in motor memory consolidation, there is behavioral, electrophysiological, and neuroimaging data indicating that motor learning is associated with somatosensory plasticity (see Ostry and Gribble [37] for review). There is also considerable evidence for the involvement of parietal cortex more generally in the control of movement [38, 39] (see Kalaska [40] for review) and motor learning [26, 41–45]. The present results indicate that this involvement extends to aspects of memory consolidation. Somatosensory areas might play a role in memory consolidation by storing newly learned sensory states that guide subsequent movements and result in improvements in somatic acuity. The finding that movements following adaptation learning are aligned with altered estimates of limb position is in agreement with this possibility [22, 46], as is the finding that somatosensory perceptual acuity increases in reinforcement and movement sequence learning [24, 25]. The storage of updated somatic information may be central to the retention of new learning. The reexpression of learned movements may require these modified states.

Consolidation in primary somatosensory cortex could also be related to its participation in the efferent control of movement and to associated learning-related cortical changes akin to those observed in primary motor cortex. Areas 3a, 3b, 1, 2, and 5 and second somatosensory cortex each send projections to the spinal cord [39, 47]. Terminations of corticospinal outputs from somatosensory cortex are most dense in the intermediate zone of the contralateral spinal cord [48], where they overlap extensively with projections from motor cortex [49]. Participation of somatosensory cortex in the efferent control of movement through terminations on spinal interneurons is suggested by the finding that identified pyramidal tract neurons discharge well in advance of the initiation of muscle activity, which suggests that they contribute to motor outflow rather than sensory input [50, 51]. The consolidation of motor memory could be associated with the stabilization of learning-related changes to the neurons that encode these outputs from somatosensory cortex.

Although motor cortex appears to have little involvement in initial motor memory consolidation, in the context of force-field learning, as shown in the present study, there are many examples of motor cortex plasticity subsequent to motor learning both in force-field tasks and in other experimental models of learning. For example, learning-related plasticity is reflected in an expanded cortical territory in motor cortex from which learned movements can be elicited [4–6, 12]. It is also seen in changes to the directional tuning of neurons in motor cortex [8, 9, 52, 53]. Since long-term potentiation of neurons in motor cortex can be produced by tetanic stimulation of somatosensory cortex [54–56], changes to motor cortex following learning could arise as an indirect effect of learning-related plasticity in somatosensory cortex. Alternatively, changes to motor cortex might be induced during the reconsolidation that occurs when motor memories are retrieved and subsequently re-stored [57]. Changes to motor cortex might also occur as a result of activity in other brain areas that have been shown to be active during learning [8, 58–63].

As in the present study, a small number of studies have applied TMS to motor cortex following learning. Any effects observed under these conditions should be attributable to consolidation and retrieval, since any possible effects on the learning process itself can be ruled out. Muellbacher and colleagues [19] applied rTMS to motor cortex after individuals learned to produce ballistic thumb–finger pinching movements and assessed effects on relearning that occurred immediately afterwards. It was found that TMS completely disrupted the retention of improvements due to learning. Baraduc and colleagues [20] replicated the Muellbacher finding but showed that interference with retention was restricted to this ballistic movement task. As in the present study, there was no interference with retention of force-field adaptation when rTMS was applied to motor cortex immediately following learning. Nevertheless, the interference with the ballistic movement task points to the involvement of motor cortex in short-term motor memory for at least some kinds of movements (cf. Robertson and colleagues, 2005 [17]). A number of studies have assessed the possibility that interference effects that are observed when retention is measured immediately after suppression of motor cortex may not prevent the eventual consolidation of learning as measured after an overnight delay. In Robertson and colleagues’ study [17], individuals learned a serial reaction-time task involving finger movement. TMS was applied to motor cortex following learning to ensure that only offline processing was affected. It was observed that when overnight consolidation of learning was permitted following rTMS to motor cortex, there was complete retention. Iezzi and colleagues [18] report a similar result. cTBS was applied over motor cortex prior to a finger-movement task. This was followed by tests of immediate retention and then a retest for retention at 24 hours. It was found that cTBS impaired retention after short periods but did not impair consolidation as measured 24 hours later.

The present study included a test to assess whether the disruption of retention seen in the somatosensory-gradual condition was due possibly to a memory retrieval failure rather than being an effect of stimulation on memory consolidation. It was found that cTBS after a 24-hour delay did not interfere with subsequent retention, which indicates that stimulation does not interfere with the retrieval of consolidated memories. This result is consistent with previous force-field learning findings that have shown a time-dependent strengthening of motor memory [64, 65]. Memories become resistant to disruption approximately 6 hours after initial learning.

Consolidation of motor memory was assessed here using an adaptation learning task, whereas much of the work to date on motor memory consolidation has used skill-learning tasks such as sequence learning. It has been shown that short-term retention of adaptation and skill learning engage different brain regions. Specifically, rTMS to primary motor cortex disrupts the retention of newly learned skills but has little effect on adaptation learning [20]. Consolidation of sequence learning in particular may rely more heavily on motor cortex than adaptation learning. For example, Robertson and colleagues [17] showed that rTMS applied to motor cortex immediately after learning disrupted retention when tested 12 hours later (without intervening sleep). One possibility is that motor cortex may play a greater role in consolidation of explicit motor skill learning, such as in sequence learning, and a reduced role in motor adaptation learning. The lack of involvement of motor cortex in the consolidation of learning in the present study may indicate that different skills and associated brain areas are engaged in explicit skills such as sequence learning and those required to compensate for abruptly introduced force-fields.

In summary, cTBS applied to somatosensory cortex following learning blocks the consolidation of motor memory and largely eliminates retention. A control study confirmed that cTBS blocked memory consolidation rather than causing a memory retrieval failure. cTBS to primary motor cortex had limited effects on retention. The failure to observe effects of stimulation on motor cortex is consistent with previous studies that show that motor cortex involvement in memory consolidation is limited, and indeed when adequate time for consolidation is permitted following stimulation, there is little evidence at all that motor cortex is involved. It is not known whether the involvement of somatosensory rather than motor cortex in motor memory consolidation is restricted to adaptation learning as shown in the present studies. However, almost all skill acquisition in adults involves some degree of adaptation, that is, of the application of known skills to new situations. In conclusion, the present study provides direct evidence that learning-related changes to somatosensory cortex are involved in the initial consolidation of motor memory. The stabilization of modified sensory states is presumably a key aspect of motor memory formation.

## Materials and methods

### Ethics statement

Procedures used in this study were approved by McGill University Faculty of Medicine Institutional Review Board (IRB study number A09-B34-12A). All participants provided written informed consent. The study adhered to the Declaration of Helsinki guidelines.

### Participants

Sixty healthy righthanded individuals (21 men, 39 women, age [mean ± SD] = 23 ± 6 years) participated in the study. Handedness was assessed using the Edinburgh handedness inventory [66]. Participants were naïve to the purpose of the experiment.

### Behavioral task

Participants held a vertical handle of a two-degree-of-freedom robotic arm (InMotion2, Interactive Motion Technologies) and made movements with the right hand in a standard point-to-point reaching task [35]. A semi-silvered mirror, which served as a display screen, was placed just below eye level and blocked vision of the arm and the robot handle (Fig 1A). Two 16-bit optical encoders provided the position of the hand (Gurley Precision Instruments) at 400 Hz. Participant-generated forces were measured using a force-torque sensor (ATI Industrial Automation) that was mounted below the robot handle. The movement start position was indicated with a white circle (20-mm diameter) 30 cm in front of the participant at the body midline. The target position, also indicated with a white circle (20-mm diameter), was 15 cm in front of the start position. The right shoulder and elbow angle at the start position were approximately 70° and 90°, respectively. The participant’s elbow was supported by an air sled.

At the start of each trial, the robot moved the participant’s arm to the start position, and after a 500-ms delay, the start position turned green, signaling the participant to initiate the movement. Participants were instructed to move to the target within 800–1,000 ms. After reaching to the target, participants were provided with color-coded feedback about their movement duration. No trials were removed for movements faster or slower than the required duration. During the movement, visual feedback of hand position was provided by a yellow cursor (5-mm diameter). Following the end of movement, the robot brought the arm back to the start position, without visual feedback of the movement path.

All participants took part in a motor learning session on day 1 and a retention and relearning session on day 2, separated by approximately 24 hours. The motor learning session began with a familiarization phase in which participants performed 20 practice movements in the absence of load. Participants were then presented with a baseline block (50 trials) in which reaching movements were performed in a null field. The baseline block was followed by a training session (150 trials) in which participants performed movements in a clockwise velocity-dependent force field. The force was applied according to the following equation:

$$\left[\begin{array}{c} f_{x}\\ f_{y} \end{array}\right]=\left[\begin{array}{cc} 0 & d\\ -d & 0 \end{array}\right]\left[\begin{array}{c} v_{x}\\ v_{y} \end{array}\right]$$ (1)

where x and y are the lateral and sagittal directions, f_x_ and f_y_ are the commanded force to the robot, and v_x_ and v_y_ are hand velocities in Cartesian coordinates. The strength of the force field was determined by the coefficient d (N.s.m^−1^), where 0 < d ≤ 15 (see Experimental groups for more detail). Six error-clamp trials were interspersed within the training block. The error-clamp trials were presented at the same position within the experimental sequence for all participants. In an error-clamp trial, the hand was constrained to move in a straight line by a stiff force channel (spring coefficient, 4,000 N/m; damping coefficient, 40 N s/m). Error-clamp trials result in movements with almost zero kinematic error, and the force applied to the channel walls during these trials provides a measure of learning. Five error-clamp trials were also inserted at the beginning of the training block to measure the baseline forces produced by the participant.

By the end of day 1, participants were able to compensate for the perturbation generated by the force field. Consolidation of this learning was assessed in a retention test that took place 24 hours later. The 24-hour delay was included to allow for consolidation and to ensure that should any loss of retention be observed, such as that seen immediately following rTMS to motor cortex [17], it is not a transient effect that is followed by subsequent recovery [18, 19]. The retention test began with five error-clamp trials. The persistence of adapted behavior in error-clamp trials (force produced in a direction opposite to the force field) reflects retention of motor memory. The error-clamp trials were followed by a relearning block, which also assesses motor memory retention. Specifically, the rate of relearning on day 2 provides a measure of savings and is thought to reflect the extent to which prior learning has been consolidated. The relearning trials took place in a force field and were the same as those in the initial training session on day 1.

### Somatosensory perception task

Measures of sensed limb position were obtained prior to and again 10 minutes after cTBS to somatosensory cortex. In the perceptual tests, the robot moved the participant’s arm straight outward either to the left or the right of the body midline. Vision of the arm was blocked. The individual was required to indicate whether the displacement of the arm was to the left or the right of the midline. The displacement direction was updated following each trial using a staircase procedure [67]. These tests were conducted using a separate group of participants (*n* = 16) to avoid any possible interference of perceptual testing with motor memory consolidation. Half of the participants were tested with sham stimulation.

### Brain stimulation

Before the force-field training session, the position at which left motor cortex was maximally excitable in eliciting MEPs in right biceps brachii was determined, using single-pulse TMS (Magstim200 stimulator). During this procedure, participants were instructed to hold the lower arm at 90° relative to gravity. The coil was placed tangentially on the scalp with the handle pointing backward and laterally at a 45° angle away from the midline. The EMG response of the biceps was recorded using Ag-AgCl surface electrodes. The active motor threshold (AMT) was defined as the minimum intensity required to elicit at least 5 MEPs (>200 μV peak-to-peak amplitude) in 10 consecutive single-pulse stimulations. To test for changes in cortical excitability after cTBS, we applied single-pulse TMS to the motor hotspot at an intensity sufficient to evoke 20 MEPs of approximately 500–700 μV (peak-to-peak amplitude) both prior to learning session and at the same intensity, 10 minutes post cTBS. The position of coil was marked and maintained by using a three-dimensional infrared optical tracking system (Polaris System, Northern Digital, Bakersfield, CA, United States) and Brainsight software (Rogue Research, Montreal, Canada).

To assess the role of motor and somatosensory cortex in the consolidation of motor memory, we used cTBS to suppress neural activity in left primary motor cortex or left somatosensory cortex immediately after learning. The site of left motor cortex stimulation was the same as that used to elicit MEPs. The site of somatosensory cortex stimulation was a point 2 cm posterior to the motor cortex stimulation position. This position overlies the postcentral gyrus [68], and magnetic stimulation at this position has been shown to change ipsilateral cortical components of somatosensory evoked potentials (SEPs) [29, 69]. cTBS was applied in two trains (10 minutes apart) of repetitive biphasic magnetic pulses (Magstim Super Rapid Stimulator) at 70% intensity of the AMT for the biceps brachii. Each train of cTBS comprised 600 pulses applied in bursts of three pulses at 50 Hz, with bursts repeated at a frequency of 5 Hz, corresponding to a total train length of 40 seconds [28, 30].

### Experimental groups

Participants were randomly assigned to one of six groups that differed based on the learning protocol and the brain area that was stimulated. The retention test and relearning session on day 2 were the same for all participants. The day 2 tests involved five error-clamp trials followed by force-field relearning with an abruptly introduced load. On the initial training (day 1), for the motor-abrupt group (*n* = 10), the force field was turned on abruptly starting at the first trial; that is, the value of d in Eq 1 was changed from 0 to 15 N.sec.m^−1^, and cTBS was applied over left motor cortex. In the somatosensory-abrupt group (*n* = 10), the force field was also introduced abruptly, but cTBS was applied over somatosensory cortex. For the somatosensory-gradual and motor-gradual groups (each *n* = 10), the force field gradually increased in strength, and cTBS was applied to somatosensory and motor cortex, respectively; the value of d in Eq 1 changed smoothly from 0 to 15 N.sec.m^−1^ over the first 135 trials. Specifically, on every trial (*n*), the strength of field (d) was calculated according to the following equation:

$$d_{n}=n^{\log\left(15\right)/\log(135)}$$ (2)

The strength of the force field remained constant for the final 15 trials, with d equal to 15 N.sec.m^-1^. A somatosensory-gradual-24h group (*n* = 10) also trained on day 1 with a gradual introduction of load. For these participants, a 24-hour delay was introduced following motor learning to permit consolidation, after which cTBS was applied to somatosensory cortex. Retention and relearning tests were conducted approximately 4 hours later. In a sham-abrupt group (*n* = 10), the onset of the force field was abrupt, and sham stimulation was applied over somatosensory cortex, with the coil placed vertically (sideways) on the scalp. This placement induced vibration and sounds that were similar to those of real cTBS stimulation, but there were no inhibitory effects.

The motor cortex stimulation data (motor-abrupt and motor-gradual) reported above are based on 20 individuals, all of whom on day 1 of testing showed suppression of MEPs following cTBS. Six additional participants did not show suppression and were excused. For those participants that showed suppression, the average suppression of MEP amplitude in comparison to baseline was 40% ± 2.9% (mean ± standard error). This ensured that any lack of effect of cTBS to motor cortex on motor memory consolidation was not due to a failure to inhibit this area. No participants in the other experimental conditions were excluded.

### Data analysis

Hand position and the force applied by the participant to the robotic arm were both sampled at 400 Hz. The position time series was low-pass filtered at 40 Hz, using a zero-phase-lag Butterworth filter, and differentiated to produce velocities. All trials were aligned with respect to peak tangential velocity, and data within ±500 ms from peak velocity were considered for further analysis. As a measure of kinematic performance during learning, the PD from a straight line between the start position and the target at maximum velocity was calculated for each movement.

The force applied to the robot arm was recorded during error-clamp trials to assess compensation for the perturbation. Baseline force profiles were subtracted from force profiles measured during error-clamp trials interspersed in the learning, relearning, and retention sessions. We quantified adaptation by regressing the ideal lateral force profile on each error-clamp trial (the force profile required for full compensation of perturbation on that trial) onto the actual force profile produced by the participant. The slope of regression line represents the relationship between the actual and the ideal compensatory force profiles and is referred as the adaptation coefficient [70]. If the applied force and the ideal force matched perfectly, the adaptation coefficient would be 1. If they are uncorrelated, it would be zero.

To compare the kinematic measures of learning across groups, we calculated the average PD over the first 10 trials of each session. ANOVA was performed on initial PD to assess differences between experimental conditions, and all post hoc comparisons were corrected by using the Holm-Bonferroni method. We calculated the rate of learning for participants that trained with abruptly introduced loads. The learning rate was calculated by robust fitting a single-rate exponential function of the form

$$y={a*exp}^{-\beta *x}+C$$ (3)

for each participant separately, where y represents the PD, a and C are constants, x represents trial number, and β is the learning rate.

To assess differences in adaptation coefficients among experimental conditions during the learning session, we compared adaptation for the final error-clamp trial with mean baseline adaptation coefficients. Mean adaptation averaged over the last two error-clamp trials was also compared across groups to assess whether all experimental groups reached similar levels of adaptation by the end of learning. Measures of retention were obtained using error-clamp trials 24 hours following initial learning. The percent retention of learning was calculated for each participant as the ratio of adaptation coefficients in the five error-clamp trials during the retention session to the mean adaptation from last two trials in the initial learning session, multiplied by 100. An ANOVA was performed on the percent retention across groups. All the post hoc pairwise comparisons were corrected by Holm-Bonferroni method for multiple comparisons. Effect sizes (ω_p_^2^ and η_p_^2^) were calculated based on methods described by Lakens [71].

### Somatosensory perception

The participant’s perception of the boundary between left and right was estimated for each participant separately by fitting a logistic function to the binary (left/right) responses to the direction of limb displacement. The distance between the 25th and 75th percentile (interquartile range) was used as a measure of perceptual acuity. A higher interquartile range indicates a poor sensitivity in the discrimination task. A mixed ANOVA was performed on interquartile range with session (pre- and post-cTBS) as a within-participant factor and TMS (somatosensory cortex and sham) as a between-participant factor.

### Removal of data

Over all participants and experimental conditions, one channel trial from the initial learning session, six channel trials from the retention session, and three force-field trials from the relearning sessions were removed. The data were removed because the participant either made a return movement midway or moved in a direction other than toward the target.

## Supporting information

S1 Data Raw data used to create summary plots in Fig 1. (XLSX)

S2 Data Raw data used to create summary plots in Fig 2. (XLSX)

S3 Data Raw data used to create summary plots in Fig 3. (XLSX)

S4 Data Raw data used to create summary plots in Fig 4. (XLSX)

## Abbreviations

cTBS: continuous theta-burst transcranial magnetic stimulation
MEP: motor-evoked potential
PD: perpendicular deviation
rTMS: repetitive transcranial magnetic stimulation
TMS: transcranial magnetic stimulation
